# Myo-inflam: a mini review on inflammatory pathways driving scleral remodeling and axial elongation

**DOI:** 10.3389/fimmu.2026.1786055

**Published:** 2026-03-27

**Authors:** Bin Lin, Wei Liang, Li-rong Cai, Meng Xu, Dong-kan Li

**Affiliations:** 1 Xiamen Eye Center and Eye Institute of Xiamen University, School of Medicine, Xiamen, China; 2 Xiamen Clinical Research Center for Eye Diseases, Xiamen, Fujian, China; 3 Xiamen Key Laboratory of Ophthalmology, Xiamen, Fujian, China; 4 Fujian Key Laboratory of Corneal & Ocular Surface Diseases, Xiamen, Fujian, China; 5 Xiamen Key Laboratory of Corneal & Ocular Surface Diseases, Xiamen, Fujian, China; 6 Translational Medicine Institute of Xiamen Eye Center of Xiamen University, Xiamen, Fujian, China

**Keywords:** axial elongation, inflammation, inflammatory mediators, myopia, scleral remodeling

## Abstract

Myopia has become a global public health challenge, with pathological high myopia leading to irreversible visual impairment. Accumulating evidence suggests that chronic inflammation is closely associated with scleral remodeling and axial elongation, the primary pathological features of myopia progression. This mini review systematically summarizes the epidemiological associations between local (ocular tissue-specific) and systemic inflammatory responses with myopia, highlighting key inflammatory mediators (NF-κB, MMP-2, IL-6, TNF-α) and their synergistic mechanisms in modulating scleral extracellular matrix degradation. We further dissect the molecular cascade of “mechanical stress - inflammation activation - ECM remodeling - fibroblast-myofibroblast transformation” that underpins myopic scleral weakening. Current research limitations include inadequate translation of animal model findings to humans, a lack of non-invasive inflammatory monitoring tools, and unstandardized biomarkers. Future directions should leverage multi-omics technologies to decode complex inflammatory networks and the gut-retina axis, facilitating the development of stage-specific precision anti-inflammatory interventions. This review offers novel insights into the inflammatory mechanisms underlying myopia and provides a theoretical basis for clinical prevention and treatment strategies.

## 1 Introduction

Myopia has emerged as a major global public health concern (1), with prevalence rising continuously, particularly reaching epidemic levels among adolescents in East Asia. Pathological changes associated with high myopia (such as macular degeneration, retinal detachment, and glaucoma) significantly increase the risk of irreversible visual impairment, imposing a heavy medical burden and socioeconomic costs.

Recent studies suggest that a chronic inflammatory response may be a key pathological mechanism underlying myopia progression. A variety of inflammatory markers, which include Nuclear Factor κB (NF-κB), Transforming Growth Factor-β (TGF-β), Interleukin-1β (IL-1β), Interleukin-6 (IL-6), Interleukin-8 (IL-8), and Tumor Necrosis Factor-α (TNF-α), exhibit abnormal expression during myopia development, and these factors may promote abnormal axial elongation by mediating scleral extracellular matrix remodeling. Experimental evidence further supports the hypothesis that anti-inflammatory drugs may slow myopia progression by regulating inflammatory pathways (2).

Current research remains controversial regarding the interactive network of core signaling pathways between inflammation and myopia, as well as the contribution of local/systemic inflammation. In terms of clinical translation, there is a lack of dynamic monitoring systems for inflammatory markers and specific targeted therapeutic strategies. This review will systematically analyze the cascade reactions of inflammatory mediators in myopic scleral remodeling, evaluate preclinical evidence of anti-inflammatory interventions, and provide a theoretical basis for the development of precise prevention and control programs based on inflammation regulation.

## 2 Epidemiological association between myopia and inflammation

### 2.1 Correlation between inflammatory markers and myopia in population studies

Population studies have demonstrated a significant association between inflammatory markers and myopia. Multiple clinical studies have detected inflammatory mediators in tear fluid, aqueous humor, and blood, revealing elevated levels of specific inflammatory factors in myopic patients, particularly those with moderate to high myopia. Among pediatric myopic populations, tear fluid analysis has shown that patients with moderate to high myopia exhibit significantly higher levels of TNF-α and intercellular adhesion molecule-1 (ICAM-1) than emmetropic individuals. Additionally, most inflammatory mediators (such as IL-1β, IL-6, and Interleukin-10) tend to increase, except for Interleukin-17 (IL-17) and vascular endothelial growth factor-A (VEGF-A) (3). Aqueous humor detection further supports this association, as soluble intercellular adhesion molecule-1 (sICAM-1) in the aqueous humor of highly myopic patients has been proposed as a potential monitoring biomarker for pathological myopia (4). Studies on blood biomarkers have also revealed a correlation between systemic inflammation and high myopia; for instance, the neutrophil-to-lymphocyte ratio (NLR), platelet-to-lymphocyte ratio (PLR), and systemic immune-inflammation index (SII) are significantly elevated in patients with high myopia (5, 6). Furthermore, assessments using the Dietary Inflammatory Index (DII) indicate that a pro-inflammatory dietary pattern may be associated with an increased risk of myopia (7). Collectively, these findings suggest a close link between local (ocular surface/intraocular) and systemic inflammatory markers and the development and progression of myopia (2, 3, 8).

### 2.2 Evidence of inflammation-induced axial elongation in animal models

Animal experiments have provided mechanistic evidence that inflammation is closely associated with axial elongation in animal myopia models. Studies have shown that myopia development is accompanied by the activation of inflammatory cascades, particularly the activation of the NF-κB signaling pathway and the upregulation of downstream pro-inflammatory factors (such as TNF-α, IL-1β, and IL-6) (2, 9). These inflammatory mediators promote matrix metalloproteinase-2 (MMP-2) expression, which contributes to scleral extracellular matrix (ECM) degradation and remodeling in experimental models. This process is associated with scleral weakening and axial elongation in form-deprivation hamster models of myopia. Notably, there is a bidirectional promotional effect between inflammation and scleral remodeling: mechanical stress (e.g., form deprivation) can trigger the initial inflammatory response, while the continuous scleral remodeling process itself further exacerbates the local inflammatory state, forming a vicious cycle of “inflammation-ECM degradation-axial elongation”. Data from the hamster models indicate that targeted inhibition of inflammatory pathways (such as the NF-κB/Matrix Metalloproteinase axis) may serve as an effective strategy to slow myopia progression (8, 9).

### 2.3 Differential impacts of local vs. systemic inflammatory responses

In myopia-related inflammatory responses, local inflammation refers to tissue-specific reactions restricted to the ocular microenvironment, including the tear fluid, aqueous humor, and scleral tissue; systemic inflammation refers to a chronic, low-grade inflammatory state of the whole body, which may be reflected in peripheral blood, lymph, and other systemic immune markers. Local inflammation plays a dominant role in myopia progression, characterized by significant changes in eye tissue-specific inflammatory markers. For example, mediators such as TNF-α and ICAM-1 in tear fluid, sICAM-1 in aqueous humor, and MMP-2 in scleral tissue have all been confirmed to be closely associated with the severity of myopia (3, 4, 8). The elevation of these local mediators is closely associated with scleral ECM remodeling and is a key factor correlated with axial elongation in human myopia (8, 9). In contrast, the association between systemic inflammation and myopia (especially high myopia) is mainly evaluated through blood biomarkers. Studies have found that systemic inflammatory indicators (such as SII, NLR, and PLR) are significantly abnormal in patients with high myopia, suggesting that a state of chronic low-grade systemic inflammation may act as a risk factor for myopia development (5, 6). C-X-C motif chemokine ligand 9 (CXCL9), C-X-C motif chemokine ligand 11 (CXCL11), and TNF receptor superfamily member 9 (TNFRSF9, also known as CD137) are also abnormally expressed in the peripheral blood of patients with high myopia (10). However, the changes in local inflammatory markers (such as tear/aqueous humor factors) are usually more significant and tissue-specific, indicating that the core pathological process of myopia progression is concentrated in the ocular microenvironment (3, 4). This difference suggests that future studies need to distinguish the biomarker profiles of local and systemic inflammation to guide personalized interventions for myopia accurately (5, 8).

To systematically summarize the epidemiological evidence linking inflammation to myopia across population studies, animal models, and distinct inflammatory response patterns, the key findings are compiled in **Table 1**.

**Table 1:** Summary of epidemiological association between myopia and inflammation.

Subcategory	Core conclusions	Key biomarkers/evidence
Population Studies	Local/systemic inflammatory markers correlate with myopia; pro-inflammatory diet increases risk.	- Local: Tear TNF-α/ICAM-1, aqueous humor sICAM-1 - Systemic: NLR, PLR, SII, plasma CXCL9/CXCL11/TNFRSF9 - Dietary: Pro-inflammatory pattern (DII)
Animal Models	Inflammation directly drives axial elongation via cascades; targeted inhibition delays myopia.	- Activated pathways: NF-κB, NLRP3-MMP-2 - Vicious cycle: Mechanical stress → inflammation → ECM degradation → axial elongation - Inhibitors: 5HPP-33, NBD peptide, MCC950
Local vs. Systemic Inflammation	Local inflammation dominates (tissue-specific, significant changes); systemic inflammation acts as a risk factor.	- Local: Ocular microenvironment-focused, strong association with myopia severity - Systemic: Chronic low-grade, linked to immune cell subset alterations

TNF, Tumor Necrosis Factor; ICAM, Intercellular Adhesion Molecule; NLR, Neutrophil-to-Lymphocyte Ratio; CXCL, C-X-C Motif Chemokine Ligand.

## 3 Mechanisms of action of core inflammatory mediators

NF-κB is a key transcription factor that regulates inflammatory responses. During myopia development, it is derepressed through the phosphorylation of Inhibitor of Nuclear Factor κB-α (IκB-α), promoting the nuclear translocation of NF-κB p50 (NF-κB1 p50)/p65 (RelA p65) subunits and activating downstream target genes (11, 12). In the form-deprivation myopia (FDM) model, the expression of phosphorylated NF-κB p65 (p-p65) in scleral tissue is significantly elevated, which is associated with the activation of the complement complex complement component 5b-9 membrane attack complex (C5b-9). The latter drives inflammatory cascades through the Toll-Like Receptor 4 (TLR4)/NF-κB axis (13). The NF-κB pathway can be activated by pro-inflammatory factors (such as TNF-α, IL-1β) and mechanical stress, thereby upregulating the expression of matrix metalloproteinases (MMPs) and interleukins (IL-6, IL-8) (12, 14, 15). Experiments have confirmed that specific inhibition of NF-κB can block TNF-α-induced secretion of MMP-2 and Metalloproteinase-9 (MMP-9) and synthesis of IL-6, which mediates the delay of scleral remodeling *in vitro* and in monocular form deprivation -induced golden Syrian hamsters models of myopia (16, 17).

MMP-2 is a key effector of scleral ECM degradation. In form-deprivation mice models, the activity and expression of scleral MMP-2 are significantly increased, a change that contributes to the degradation of type I collagen (Col-I) and is associated with impaired scleral biomechanical strength (11, 18, 19). Its activation mechanisms include: (1) NF-κB pathway-dependent transcriptional upregulation, such as TNF-α enhancing MMP-2 promoter activity via TNF receptor 1 (TNFR1) (15); (2) regulation by the NLR family pyrin domain containing 3 (NLRP3) inflammasome, where the NLRP3 inhibitor 4-(2-(4-Fluorophenyl)-2-oxoethyl)-1-(methylsulfonyl)piperidine-4-carboxamide (MCC950) can reduce MMP-2 expression and increase Col-I deposition (20); (3) paracrine action of the myokine IL-6, and *in vitro* experiments have confirmed that IL-6 released by skeletal muscle contraction can upregulate MMP-2 expression in scleral fibroblasts (21). Additionally, the concentration of MMP-2 in the aqueous humor of myopic patients is positively correlated with axial length (MMP-2 in high myopia patients: 13.10 ng/mL vs. non-high myopia: 8.82 ng/mL, P = 0.0003) (22, 23).

IL-6 and TNF-α synergistically amplify inflammatory signals through multiple pathways:

IL-6: Activates the Pathway: Janus Kinase/Signal Transducer and Activator of Transcription (JAK/STAT) pathway in scleral fibroblasts via the trans-signaling pathway (IL-6R/gp130 complex), promoting MMP-2 secretion. In lens-induced myopia guinea pig models, overexpression of scleral IL-6 is associated with axial elongation, while IL-6 receptor antagonists (e.g., olamkicept) can block this effect. Bioinformatics analysis shows that the expression of Interleukin-6 Signal Transducer, a core component of the IL-6 signaling pathway, is upregulated in myopic tissues (21, 24).

TNF-α: Activates the mitogen-activated protein kinase (MAPK)/NF-κB cascade via TNFR1, stimulating scleral cells to release matrix metalloproteinase-1 (MMP-1), matrix metalloproteinase-3 (MMP-3), and pro-inflammatory factors (IL-1β, IL-8) (12, 14, 15). Clinical studies have shown that the level of TNF-α in the aqueous humor of highly myopic patients is significantly elevated and positively correlated with axial elongation (22, 23, 25).

The two form a positive feedback loop: TNF-α enhances NF-κB-mediated IL-6 transcription, while IL-6 further activates signal transducer and activator of transcription 3 (STAT3) to maintain the continuous production of TNF-α, collectively driving scleral ECM remodeling (12, 14, 26, 27).

The core inflammatory mediators discussed exhibit well-documented protein-protein interactions that underpin their synergistic roles in scleral remodeling. TNF-α directly activates NF-κB via TNFR1, while IL-6 binds to IL-6R/gp130 to trigger STAT3 signaling; together, these two cytokines form a positive feedback loop that amplifies inflammation. NLRP3 inflammasome activation directly or indirectly upregulates MMP-2 expression, and NF-κB further enhances MMP-2 transcription, collectively mediating collagen degradation. This known interaction network highlights the systematic crosstalk driving myopic scleral remodeling.

**Table 2** synthesizes the activation triggers, downstream biological effects, and potential regulatory targets of the core inflammatory mediators (NF-κB, MMP-2, IL-6, and TNF-α) involved in myopia progression, providing a concise overview of their mechanistic roles.

**Table 2:** Summary of mechanisms of action of core inflammatory mediators.

Mediator	Activation triggers	Downstream effects	Regulatory targets/inhibitors
NF-κB	Pro-inflammatory factors (TNF-α/IL-1β), mechanical stress, complement C5b-9 (TLR4 axis)	Nuclear translocation of p50/p65; upregulates MMPs 2&9 and IL-6&8	5HPP-33, NBD peptide
MMP-2	NF-κB pathway, NLRP3 inflammasome, IL-6 (paracrine), hypoxic microenvironment (HIF-1α/HIF-2α)	Degrades scleral Col-I; reduces biomechanical strength; correlates with axial length	MCC950 (NLRP3 inhibitor)
IL-6	Mechanical stress-induced myokine release, hypoxic environment	Activates JAK/STAT pathway; promotes MMP-2 secretion; induces axial elongation	Olamkicept (IL-6 receptor antagonist)
TNF-α	Mechanical stress, inflammatory cascade amplification	Activates MAPK/NF-κB; stimulates MMP-1/3 and IL-1β/8 release; enhances IL-6 transcription	Indirect inhibition via NF-κB blockers

NF-κB, Nuclear Factor κB; TNF, Tumor Necrosis Factor; IL, Interleukin; MMP, Matrix Metalloproteinase; NLRP3, NLR Family Pyrin Domain Containing 3.

## 4 Molecular cascade of inflammation-scleral remodeling

Axial elongation during myopia progression is closely associated with the structural remodeling of the scleral ECM, a process precisely regulated by inflammatory signaling cascades. The molecular mechanisms are elaborated below from three key nodes: mechanical stress initiation, matrix metalloproteinase-mediated collagen degradation, and fibroblast phenotypic transformation.

### 4.1 Mechanical stress-induced inflammatory initiation mechanism

Mechanical stress exerted on the sclera (e.g., changes in intraocular pressure) is the initiating factor of inflammatory cascades. Studies have shown that mechanical strain promotes the upregulation of TGF-β1 expression by activating the mechanosensitive protein YES-associated protein (YAP) in scleral fibroblasts, thereby contributing to collagen metabolic disorders in experimental models (28, 29). In glaucoma models, mechanical strain induced by increased intraocular pressure can trigger the migration and transcriptional reprogramming of scleral fibroblasts, activating the TGF-β1/Sma- and Mad-related protein 3 (Smad3) signaling axis and ultimately contributing to ECM remodeling (30–33). Additionally, single-cell RNA sequencing has confirmed that mechanical stress regulates the expression of α-smooth muscle actin (α-SMA) through the YAP pathway, promoting the transformation of fibroblasts into myofibroblasts and accelerating scleral structural weakening (34).

### 4.2 Metalloproteinase activation and collagen degradation pathways

MMP-2 is the core effector molecule mediating scleral collagen degradation. Its activation is strictly regulated by the NLRP3 inflammasome: NLRP3 activation promotes MMP-2 upregulation in scleral tissue, which in turn mediates the degradation of Col-I—the major structural component of the sclera—resulting in disorganized collagen fiber arrangement and widened inter-fiber spaces (*in vivo* evidence from both lens-induced myopia guinea pig models and form-deprivation myopia mouse models) (13, 20, 35). Animal experiments have confirmed that the use of the NLRP3 inhibitor MCC950 can significantly reduce MMP-2 activity, which mediates the inhibition of collagen degradation and the attenuation of myopia progression in mouse models (20). Meanwhile, the hypoxic microenvironment synergistically enhances the transcriptional activity of MMP-2 by inducing the expression of hypoxia-inducible factors (HIF-1α/HIF-2α), forming a positive feedback loop of “hypoxia-MMP-2-ECM degradation” (23, 24).

Beyond hypoxia, other stressors—including oxidative stress and metabolic stress—may also converge with inflammatory signaling to regulate scleral remodeling. Oxidative stress-induced reactive oxygen species (ROS) can activate NLRP3 inflammasome and NF-κB pathways, further amplifying MMP-2-mediated collagen degradation in myopic scleral tissue (8, 36). Metabolic stress, such as nutrient imbalance or impaired mitochondrial function, may exacerbate scleral fibroblast dysfunction and reduce type I collagen synthesis, synergizing with hypoxia to weaken scleral biomechanical strength (37). Notably, hypoxia and these stressors do not act independently but form a “stress-inflammation-remodeling” cascade: stress-induced cellular damage triggers local inflammation, which in turn accelerates ECM degradation. While persistent remodeling further aggravates the hypoxic/stress microenvironment (38, 39). This integrated crosstalk highlights the multi-factorial nature of myopic scleral remodeling, extending beyond isolated inflammatory pathways.

### 4.3 Key nodes of scleral fibroblast phenotypic transformation

The phenotypic transformation of fibroblasts into myofibroblasts serves as the structural basis of scleral remodeling. The core of this transformation is coordinately regulated by the TGF-β1/Smad3 and YAP pathways:

TGF-β1/Smad3 axis: Mechanical strain activates TGF-β1 expression via YAP, and the latter phosphorylates Smad3 protein, promoting the transcription of α-SMA and collagen synthesis-related genes and contributing to myofibroblast differentiation in experimental models (29, 31).

Inflammatory factor synergy: IL-6 is associated with increased fibroblast proliferation and differentiation under hypoxic conditions *in vitro*, while being correlated with reduced cell apoptosis; these changes are associated with scleral thinning in human myopia (40).

Phenotypic marker: Overexpression of α-SMA is a direct marker of phenotypic transformation, and its combination with reduced type I collagen synthesis collectively leads to a decrease in scleral biomechanical strength (28, 41). Inhibition of YAP or Smad3 can effectively block this transformation process *in vitro*, providing potential targets for targeted interventions in clinical settings (31, 34).

In summary, the cascade reaction of “mechanical stress - inflammation - ECM degradation - cellular phenotypic transformation” constitutes the core molecular framework of myopic scleral remodeling.

## 5 Current challenges and future directions

### 5.1 Methodological limitations in mechanistic research

Current research on the mechanisms underlying inflammation and myopia progression has significant methodological limitations. The pathophysiological processes of animal models differ from those of human myopia, which limits the clinical translational value of their research findings (42). Insufficient monitoring technologies make it difficult to assess the dynamics of local ocular inflammation in humans in a real-time and non-invasive manner (e.g., aqueous humor cytokine levels). Most existing methods rely on invasive sampling (such as aqueous humor paracentesis), which cannot meet clinical needs (43, 44). Molecular mechanism studies mostly focus on single pathways (e.g., NF-κB, MMP-2), lacking systematic analysis of multi-factor synergistic networks (such as the IL-6/TNF-α/NF-κB interaction). Additionally, *in vitro* cell experiments are difficult to simulate the complexity of the *in vivo* microenvironment (31, 42, 45).

### 5.2 Bottleneck issues in clinical translation

The clinical translation of anti-inflammatory therapeutic strategies faces multiple bottlenecks. Atropine, as an effective drug for slowing myopia progression, has a mechanism of action (especially the anti-inflammatory pathway) that remains unclear, leading to difficulties in dose optimization and side effect management (46–49). Candidate drugs targeting the NF-κB/MMP pathway (e.g., MCC950) lack large-scale clinical trials to support their long-term safety and feasibility of human formulations (42, 50, 51). The detection standardization of inflammation-related biomarkers (e.g., CXCL9, CXCL11, TNF-α) is insufficient, and their critical values for predicting myopia progression have not been established, hindering personalized interventions (52–54). Although existing anti-inflammatory interventions (e.g., diacerein, lactoferrin) have shown potential in experimental models, the evidence for clinical efficacy is weak, and the regulatory impact of systemic inflammatory states (e.g., gut microbiota dysbiosis) on local ocular tissues has not been considered (55–57).

### 5.3 Prospects of multi-omics integration and precision intervention pathways

Future research needs to integrate multi-omics technologies to overcome mechanistic and translational bottlenecks. Through combined genome-transcriptome-proteome analysis (e.g., UK Biobank cohort), myopia-specific inflammatory network nodes can be identified, and novel theoretical models such as the “muscle-myokine-scleral remodeling” axis can be constructed (58–60). Utilizing single-cell sequencing to resolve the immune-micro-environment heterogeneity of the retina/sclera in high myopia (e.g., macrophage subsets, T cell infiltration) can reveal the crosstalk between aging, immunometabolism, and inflammation (61–63). Microbiome-metabolome association studies can clarify the “gut-retina axis” mechanism (e.g., metabolite changes induced by gut microbiota dysbiosis affecting intraocular inflammation), providing targets for probiotic/dietary interventions (34, 55, 57, 64). Risk prediction models based on multi-omics data (e.g., Mendelian randomization analysis) can identify high-risk populations (65, 66) and guide precise anti-inflammatory strategies: developing IL-6 trans-signaling inhibitors for early-stage myopia (59); designing NLRP3-MMP-2 pathway antagonists for rapid progression stages (42, 45); and optimizing biomarker combinations (e.g., tear TNF-α + serum CXCL9) with artificial intelligence to achieve dynamic monitoring (53, 54, 67).

## 6 Conclusion

This review systematically synthesizes the bidirectional crosstalk between inflammation and myopia progression, highlighting inflammation as a core pathological driver of scleral remodeling and axial elongation. Population studies confirm consistent associations between local (tear/aqueous humor TNF-α, sICAM-1, MMP-2) and systemic (NLR, PLR, CXCL9) inflammatory markers with myopia severity, while animal models demonstrate that inflammatory cascades (e.g., NF-κB, NLRP3-MMP-2 axis) mediate scleral extracellular matrix degradation in experimental models. The molecular cascade, initiated by mechanical stress in experimental models, is amplified via IL-6/TNF-α signaling crosstalk, which is correlated with both processes and culminates in fibroblast-myofibroblast transformation associated with scleral biomechanical weakening, establishing a clear framework linking inflammation to scleral biomechanical weakening.

Despite these advances, critical gaps remain: methodological limitations in translating animal model findings to humans, inadequate non-invasive inflammatory monitoring tools, and a lack of standardized biomarkers hinder clinical translation. Future research should leverage multi-omics technologies to decode complex inflammatory networks and the gut-retina axis, while developing stage-specific precision interventions (e.g., IL-6 trans-signaling inhibitors, NLRP3 antagonists). By integrating mechanistic insights with clinical utility, targeted anti-inflammatory strategies hold promise for addressing the global myopia epidemic, reducing irreversible visual impairment, and alleviating associated socioeconomic burdens.

## Glossary

5HPP-33: 5-(4-Hydroxyphenyl)-3-phenyl-1H-pyrazole-4-carboxamide
α-SMA: α-smooth muscle actin
C5b-9: complement component 5b-9 membrane attack complex
Col-I: type I collagen
CXCL9: C-X-C motif chemokine ligand 9
CXCL11: C-X-C motif chemokine ligand 11
DII: Dietary Inflammatory Index
ECM: Extracellular Matrix
FDM: Form-deprivation myopia
HIF-1α: Hypoxia-inducible factor-1α
HIF-2α: Hypoxia-inducible factor-2α
ICAM-1: Intercellular Adhesion Molecule-1
IL-1β: Interleukin-1β
IL-6: Interleukin-6
IL-6R: Interleukin-6 Receptor
IL-8: Interleukin-8
IL-10: Interleukin-10
IL-17: Interleukin-17
IκB-α: Inhibitor of Nuclear Factor κB α
JAK/STAT: Janus Kinase/Signal Transducer and Activator of Transcription
MAPK: Mitogen-Activated Protein Kinase
MMP-1: Matrix Metalloproteinase-1
MMP-2: Matrix Metalloproteinase-2
MMP-3: Matrix Metalloproteinase-3
MMP-9: Matrix Metalloproteinase-9
NBD: Neutrophil Band Density
NF-κB: Nuclear Factor κB
NLR: Neutrophil-to-Lymphocyte Ratio
NLRP3: NLR Family Pyrin Domain Containing 3
NLRP3-MMP-2 Axis: NLRP3 Inflammasome-Matrix Metalloproteinase-2 Axis
PLR: Platelet-to-Lymphocyte Ratio
SII: Systemic Immune-Inflammation Index
Smad3: Sma- and Mad-related protein 3
STAT: Signal Transducer and Activator of Transcription
STAT3: Signal Transducer and Activator of Transcription 3
TGF-β: Transforming Growth Factor-β
TGF-β1: Transforming Growth Factor-β1
TLR4: Toll-Like Receptor 4
TNF: Tumor Necrosis Factor
TNF-α: Tumor Necrosis Factor-α
TNFR1: TNF Receptor 1
TNFRSF9: TNF Receptor Superfamily Member 9 (CD137)
VEGF-A: Vascular Endothelial Growth Factor-A
YAP: YES-associated protein
sICAM-1: Soluble Intercellular Adhesion Molecule-1
